# Viruses Run: The Evasion Mechanisms of the Antiviral Innate Immunity by Hantavirus

**DOI:** 10.3389/fmicb.2021.759198

**Published:** 2021-09-30

**Authors:** Yusi Zhang, Ruixue Ma, Yutong Wang, Wenjie Sun, Ziwei Yang, Mingwei Han, Tixin Han, Xing-an Wu, Rongrong Liu

**Affiliations:** ^1^Department of Immunology, School of Basic Medicine, Fourth Military Medical University, Xi΄an, China; ^2^Department of Microbiology, School of Basic Medicine, Fourth Military Medical University, Xi΄an, China; ^3^School of Basic Medicine, Fourth Military Medical University, Xi΄an, China

**Keywords:** hantavirus, immune evasion, IFN, PRR, cell death, antiviral innate immunity

## Abstract

Hantavirus can cause hemorrhagic fever with renal syndrome (HFRS) in Eurasia and hantavirus pulmonary syndrome (HPS) in America, with high mortality and unknown mechanisms. Innate immunity is the host’s first-line defense to bridge the acquired immunity against viral infections. However, hantavirus has evolved various strategies in both molecular and cellular aspects to evade the host’s natural immune surveillance. The Interferon-I (IFN-I) signaling pathway, a central link of host defense, induces various antiviral proteins to control the infection. This paper summarizes the molecular mechanisms of hantavirus evasion mechanisms of the IFN signaling pathway and cellular processes such as regulated cell death and cell stress. Besides, hantavirus could also evade immune surveillance evasion through cellular mechanisms, such as upregulating immune checkpoint molecules interfering with viral infections. Understanding hantavirus’s antiviral immune evasion mechanisms will deepen our understanding of its pathogenesis and help us develop more effective methods to control and eliminate hantavirus.

## Brief Introduction of Hantavirus

In recent years, the repeated outbreaks of diseases caused by hantavirus have seriously threatened human health. Hantavirus syndrome is caused by hantaviruses infection and is a type of emerging zoonosis. Hantavirus has been found in Europe, Asia, North America, and parts of South America. It is a pathogen sometimes transmitted from animals to humans. Through gene sequence alignment and serodiagnosis, hantaviruses are categorized into more than 20 types; some have different geographical distributions and unique rodent hosts. Old World Hantavirus, including Hantaan virus (HTNV), Dobrava virus (DOBV), Puumala virus (PUUV), and Seoul virus (SEOV), mainly targets human kidneys, causing hemorrhagic fever with renal syndrome (HFRS). New World Hantavirus, including Sin Nombre virus (SNV) and Andes virus (ANDV), mainly targets the lungs, causing hantavirus cardiopulmonary syndrome (HCPS). However, more and more studies have shown that many clinical manifestations overlap between HFRS and HCP (Clement et al., 2019). Hantavirus is mainly transmitted by rodents, which pollute the surrounding food and water sources through their excreta and saliva. It then spreads through the respiratory tract, digestive tract, and skin damage caused by biting and scratching (Clement et al., 2019). It is also reported that the virus can be transmitted vertically from mother to child. The clinical symptoms of infected pregnant women and their fetuses will be more severe, and the prognosis will worsen (Lu et al., 2021). Compared with adults, children have a milder course of hantavirus infection (Echterdiek et al., 2019). People susceptible to infection mainly include field workers, outdoor explorers, agriculture, construction workers, and rodent veterinarians easily exposed to rodent excrement (Duggan, 2019). Rodent control and public health education and promotion play a significant role in preventing hantavirus infection (Dheerasekara et al., 2020).

Hantavirus is in the Bunyaviridae family. Elliott and McGregor (1989) determined the complete genome sequence of the Bunyaviridae family (Elliott, 1989). All members of the Bunyaviridae share a similar genome structure. Hantaviruses are enveloped, negative-sense, single-stranded RNA viruses (ssRNA), with three segments named small (S), medium (M), and large (L; Elliott, 1990). L segment encodes the viral RNA-dependent RNA polymerase (RdRp), while M and S segments encode the precursor (GPC) for two viral surface glycoproteins (GnP and GcP), and the nucleocapsid protein (NP), respectively (Elliott, 1990). The S segment of many bunyaviruses also encodes non-structural proteins (NSs; Hart et al., 2009).

The incubation period of HFRS is approximately 12–16days; however, this can vary from 5 to 42days. Typical HFRS clinical courses include five phases: febrile, hypotensive, oliguric, diuretic, and convalescent. Stages may occasionally overlap (Liu et al., 2021). Although hantavirus infections have a worldwide distribution with a high mortality rate, there are no currently safe and effective vaccines or therapeutics for hantavirus-related diseases (Engdahl and Crowe, 2020; Liu et al., 2020). Endothelial cells and monocyte/macrophages are generally considered the principal target cells of hantaviruses (Sacks et al., 2018). However, recent breakthroughs have been made in the pathogenic mechanism of HTNV. Liu et al. (2021) showed for the first time that a significant portion of CD8^+^ T cells in patients at the acute phase of severe HFRS harbored HTNV nucleocapsid protein, and then confirmed that primary human CD8^+^ T cells were not only permissive to HTNV infection *in vitro* but also supported the complete viral replication cycle using electoral microscopy. The study revealed a cross-talk between virus and host factors and suggested some observed changes related to innate immune response. Exemplified by the current COIVD-19 pandemic, zoonotic infections can cause havoc to human society. We reasoned that lessons learned from studying a specific zoonotic viral infection, hantavirus infection, can be helpful in the understanding of virus-host interaction in general.

## The Latest Researches Related to Innate Anti-Hantavirus Immune Responses

The innate immune system, characterized by interferon (IFN) responses and innate immunocyte activation, provides the first line of defense against hantavirus infection. IFN signaling is activated by different mechanisms. It has been demonstrated that the virus RNA rather than the virion proteins acts as pathogen-associated molecular patterns (PAMPs) to trigger innate immune activation during hantavirus infection (Zhang et al., 2014; Kell et al., 2020). In the significant target cells, like endothelial cells and epithelial cells, hantavirus was recognized by RNA helicase retinoic acid-inducible gene I (RIG-I; Lee et al., 2011; Zhang et al., 2014; Kell et al., 2020), TLR3 (Handke et al., 2009; Zhang et al., 2014), and MDA5 (Zhang et al., 2014) and induced the following interferon signaling pathway.

Nucleotide-binding oligomerization domain-like receptors (NLRs) were also reported to be involved in anti-hantavirus infection. Ye et al. (2015) described that in the THP-1 cells, the formation of the NLRP3 inflammasome was responsible for the induction of IL-1β. NLRC3 is a negative regulator, which attenuates the reaction of type I interferon (IFN-I) by isolating and attenuating the stimulation of the interferon gene (STING). Ma et al. (2021) have shown that after attacking the virus, NLRC3^−/−^ mice can show symptoms similar to patients characterized by thrombocytopenia, renal tubular dilatation, and hemorrhage, which can be a potential disease research model. Besides the classical pathogen recognition receptors (PRRs), long noncoding RNAs (lncRNAs), and miRNAs also regulate innate immunity. Ma et al. (2017) identified that the lncRNA NEAT1 served as a positive modulator for RIG-I signaling and acted antiviral function in endothelial cells. MiR-145-5p, packaged into exosomes released from hantavirus infected endothelial cells, was found to transmit the signal to the recipient cells, induce the type I interferon response, and inhibit hantavirus infection (Wang et al., 2020). The innate immunocytes were another weapon to defend against hantavirus infection. Dendritic cells infected by hantavirus produced pro-inflammatory cytokines, such as TNF-α and IFN-α, and activated T cells efficiently (Raftery et al., 2002). Monocytes infected by hantavirus resulted in activation of the oxygen-dependent metabolism and NO-synthase, which were correlated with its phagocytosis activity (Plekhova et al., 2005). Monocytes exposed to PUUV also induced IFN-α and MxA production to mediate resistance to this virus (Temonen et al., 1995). Furthermore, a recent study has described that primary monocytes and endothelial cells infected by PUUV could even activate mucosal-associated invariant T (MAIT) cells, which might increase the cytolytic potential of MAIT cells and exert its antiviral effect (Maleki et al., 2021). Understanding the anti-virus immune escape mechanisms of hantavirus will deepen our understanding of the pathogenesis of hantavirus and help us develop more effective methods to control and eliminate hantavirus.

## Molecular Mechanisms of Hantavirus to Evade Innate Antiviral Immune Responses

### Virus Proteins Inhibit IFN Signaling

Virus-induced interferon expression is not only a critical part of the innate cellular immune response but also the first defense against virus invasion, thus limiting viral replication (Samuel, 2007). Studies have shown that pathogenic New World Hantavirus and non-pathogenic hantavirus modulate the innate immune response and evade interferon-mediated antiviral signals in different ways (Smith and Ward, 2006). It has been confirmed that pathogenic New York-1 virus (NY-1V) and HTNV replicate in human endothelial cells and regulate early IFN response (Alff et al., 2006). Co-expression of the NY-1V Gn cytoplasmic tail inhibited TBK-1-directed IFN-β transcriptional responses and inhibited RIG-I-directed IFN-stimulated response elements (ISRE) transcription. Their following study illustrated that the NY-1V Gn tail interacted with TRAF3 and disrupted the formation of the TBK1-TRAF3 complex, which affected the IFN-β transcription and then prevented IFN-β induction (Alff et al., 2008). In 2014, the same group demonstrated that besides the Gn proteins from NY-1V, Gn proteins of ANDV and Tula virus (TULV) containing elements in their 142-residue cytoplasmic tails could inhibit RIG-1/mitochondria antiviral signaling protein (MAVS)/TBK-1-TRAF3-directed IFN-β induction by binding with TRAF3 in the TRAF-N domain (Matthys et al., 2014). Furthermore, as with NY-1V, SEOV infection damped the antiviral responses and dramatically suppressed the IFN-β induction (Au et al., 2010). In ANDV infection, both NP and GPC were found to inhibit the induction of IFN-β and block the downstream JAK/STAT signaling (Levine et al., 2010). Although NP’s precise inhibition mechanism remains to be clarified, Levine et al. (2010) speculated that the NP of hantavirus might interact with proteins responsible for the posttranslational modification, such as small ubiquitin-related modifier 1 (SUMO-1). Besides the NP and GP, the ANDV NSs protein also has suppressive properties to modulate the immune response. By interacting with MAVS, ANDV-NSs protein suppressed IFN-β promoter activity when the signaling pathway was activated by ectopic expression of MDA5, RIG-I, or TBK1. However, their study did not unravel the precise molecular mechanism on how ANDV-NSs protein inhibited the MAVS signaling pathway. Further studies are still needed (Vera-Otarola et al., 2020). Similar results were seen during PUUV infection. GPC and NSs proteins of PUUV were found as inhibitors to suppress RIG-I-mediated IFN-β production and ISRE activation (Gallo et al., 2021).

In contrast to pathogenic hantaviruses, the non-pathogenic hantaviruses-Prospect Hill (PHV) elicits robust interferon response by inducing IRF-3 activation early after infection within human endothelial cells simultaneously. It directs high-level ISG56 and MxA compared with NY-1V or HTNV, and the degree of STAT-1/2 phosphorylation in PHV-infected cells was considerably higher than that in ANDV (Smith and Ward, 2006), which are in line with reduced PHV replication (Alff et al., 2006). However, as for TULV, low-pathogenic hantaviruses replicate successfully in human endothelial cells, suggesting that TULV can regulate cellular IFN responses. Alff et al. (2006) pointed out that expression of the cytoplasmic tail of TULV Gn protein suppressed IFN response at the level of regulating TBK1-directed transcriptional ISRE and IFN-β responses, which was the same as NY-1V, yet TULV protein was unable to bind TRAF3 (Alff et al., 2008).

### Cell Death During HTNV Infection

Hantavirus escapes the immune response by regulating cell death, mainly including autophagy, apoptosis, and pyroptosis. Cells and organisms can utilize autophagy to remove damaged organelles, degrade toxic proteins, and achieve bioenergetic materials recycling, which is necessary for survival (Li et al., 2021a). With constant evolution, some viruses gained a capacity to hijack, evade, or manipulate the host autophagy process to complete their life cycle (Mao et al., 2019). Muhammad et al. found that SNV Gn can be rapidly degraded by autophagy to decrease the intrinsic steady-state levels in the early replication and assembly stages, indicating that autophagy clearance of Gn is necessary for efficient hantavirus replication (Hussein et al., 2012; Ganaie and Mir, 2014). Wang et al. (2019) showed that the HTNV could hijack the host autophagy machinery to manipulate a complete mitophagy at the early replication stage and incomplete autophagy at the packaging and assembly stage. Gn-induced mitosis promoted MAVS degradation and delayed host IFN response. NP prevents the autophagy-dependent clearance of Gn by binding to LC3B and SNAP29. Inhibition of autophagy in the early stage of infection can limit HTNV replication (Wang et al., 2019).

There are accumulating studies showing that hantavirus infection inhibits apoptosis in the infected cell through extrinsic or intrinsic pathways to support viral replication and survival. For example, Jonas Klingström et al. demonstrated that six different orthohantaviruses all showed an inhibitory effect of apoptosis and function of cytotoxicity of lymphocyte on infected cells, and the NP of different orthohantaviruses can inhibit granzyme B and caspase-3 activity (Solà-Riera et al., 2019a). In addition, hantaviruses could inhibit apoptosis of infected cells in an intrinsic manner that manifests at the mitochondrial level by upregulating the pro-survival factor BCL-2 expression and subsequent activation caspases 3, 8, and 9 (Solà-Riera et al., 2020). They also proved that by promoting ubiquitination of death receptor 5 (DR5), hantavirus inhibited TNF-related apoptosis-inducing ligand (TRAIL)-mediated extrinsic apoptosis induction in infected cells (Solà-Riera et al., 2019b). It is also showed that the NP of TULV inhibited apoptosis by binding with, and sequestered caspase-3C (Davies et al., 2019).

However, some researches showed that hantaviruses could also induce apoptosis in some cell lines. Whether induction of apoptosis is for virus evasion of host anti-viral effect is still inconclusive. For example, cultured Vero E6 cells exhibited characteristic features of apoptosis, including condensation and segmentation of nuclei and internucleosomal cleavage of nuclear DNA when infected by the HTNV or the PHV (Kang et al., 1999). Hantavirus infection also induced apoptosis in human embryonic kidney cell line HEK293, which might be linked to the persistence and pathogenesis in hantavirus infections (Markotic et al., 2003). Xu et al. (2005) demonstrated that under the influence of cordycepin (Cor), HTNV infection of the human embryonic pulmonary fibroblasts (HEPF) could induce apoptosis by detecting caspase-3 activity, annexin V binding, and cell cycle. Their results also indicated that with the induction of apoptosis, a reduced and slowed viral maturation occurred in HEPF. Another study showed that the NP and GP of HTNV could induce TRAIL expression in HUVECs and promote cells apoptosis, leading to IFN-β production and exhibiting an antiviral effect (Chen et al., 2020). Further studies are needed to reveal the role of apoptosis in hantavirus target cells.

Only one study has reported pyroptosis associated with hemorrhagic fever disease, but that was in Zebrafish Larvae. Using zebrafish larvae as a viral hemorrhagic diseases model, Varela et al. (2014) found that pyroptosis and IL-1β release could be observed in the macrophages after rhabdovirus spring viremia of carp virus (SVCV) infection. Ye et al. (2015) reported that HTNV induces the formation of the NLRP3 inflammasome in THP-1 cells and this may be responsible for the elevated IL-1β levels in HFRS patients. It has been reported in the literature that after the inflammasome is activated, caspase1 cleaves IL-1β and IL-18 precursors into mature forms, and cleaves Gasdermin D (GSDMD) to induce cell membrane perforation and pyroptosis (Pan et al., 2021). However, whether hantavirus causes pyroptosis or not needs further study.

The ability of the virus to cause cell death is directly related to its pathogenicity. Studying whether and how cells die after virus infection is important for us understanding the interaction between virus and cells and host immunity and providing unique insights for potential therapeutic intervention. In 2019, Kanneganti et al. proposed the concept of “PANoptosis.” PANoptosis is an interplay of three different cell death pathways – pyroptosis, apoptosis, and necroptosis. When pathogens or other blockers destroy one or more programmed death pathways, PANoptosis provides an alternative cell death defense mechanism for the host to coordinate adaptive immune responses to promote pathogen elimination (Christgen et al., 2020; Malireddi et al., 2020; Samir et al., 2020). In turn, inflammation is triggered by cell death, leading to the release of more cytokines and inflammatory molecules, which are closely related to SARS-COV2, MERS, IAV, and other viral infectious diseases (Malireddi et al., 2019; Christgen et al., 2020; Lee et al., 2020). Based on some researches, we speculate that “PANoptosis” would also occur after hantavirus infects different kinds of cells, which needs further researches.

### miRNA Released From Virus Target Cells Promote Virus Infection

The link between miRNAs and viruses has been shown in recent studies. miRNA released from virus target cells is reported to regulate virus infection and replication. Studies have found that HTNV infection and HTNV NP/GP can promote the production of miR-146a in human umbilical vein endothelial cells (HUVECs), which can negatively regulate the NF-κB pathway. Therefore, using miR-146a mimic could reduce the expression of pro-inflammatory cytokines, thus escaping the host immune response. It was also discovered that viral proteins (NP/GP) could increase the transcriptional activity of the miR-146a promoter (Chen et al., 2017). Latest research reports that RNA sequencing of HTNV infection and mock infection HUVEC showed that in the process of HTNV infection, a total of 70 circRNAs, 66 miRNAs, and 788 mRNAs were differentially expressed by promoting or inhibiting virus replication (Lu et al., 2020). These studies give us a hint regarding the small RNA as a novel therapeutic target for HTNV infection.

### Virus-Induced Cell Stress

Virus infection could induce cell stress in both target cells and lymphocytes. However, whether the hantavirus would benefit from cells is still controversial. It might be relevant to different cell models. The study confirmed that TULV infection caused ER stress, which mediated the death program in Vero E6 cells (Li et al., 2005). They thought that ER stress might cause the production of pro-inflammation cytokines. Similarly, recent research reported that HTNV infection could induce ER stress in differentiated THP-1 (dTHP-1) cells (Li et al., 2021b). They believed that ER stress is the process of self-compensation and self-protection. In HUVECs, Christ et al. (2020) found that hantavirus infection could inhibit stress granule formation mediated by protein kinase R (PKR) and PKR-like ER kinase (PERK), which sensitively respond to cell stress. These mechanisms help the hantavirus escape the detrimental effects of host stress signaling (Christ et al., 2020). The role of the induction of cell stress in different cells needs further exploration.

## Cellular Mechanisms of Hantavirus to Evade Innate Antiviral Immune Responses

The battle between viruses and host cells is complex and fierce. Au et al. (2010) infected both dendritic cells (DCs) and macrophages with SEOV and found that the expression of MHC-II, CD80, IL-6, IL-10, TNF-α, and IFN-β was reduced. Their results indicated that hantavirus infection suppressed the innate immune response potential of antigen-presenting cells (APCs), which connected the adaptive immune response to the innate immune response. Li et al. stated that SEOV infection could also increase the level of PD-L1 (Li and Klein, 2012). They infected lung microvascular endothelial cells (LMVECs) of Norway rats with SEOV. SEOV infection failed to induce antiviral pro-inflammatory cytokines and promoted the expression of TGF-β and PD-L1 in LMVECs. These may help the SEOV to replicate and evade immune surveillance. Raftery et al. (2018) described an imperfect immune evasion of hantavirus. Hantavirus infection induced surface expression of PD-L1 and PD-L2 on the endothelial cells and monocyte-derived DCs. The upregulation of PD-L1 and PD-L2 could have been used as a way to evade immune surveillance. However, their study found that costimulatory markers on DCs, such as CD80 and CD86, were also upregulated. These caused bystander CD8^+^T cells activation is bypassing the checkpoint inhibition. However, they did not test the cytokines production and cell killing ability of the activated T cells in their study. The outcome of their battle needs to be further determined.

## Future Perspective

**Figure 1:**
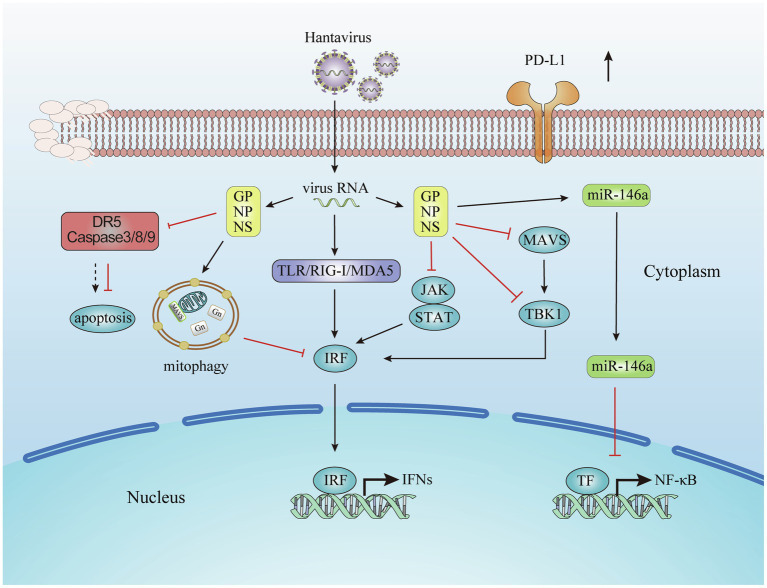
Evasion of the innate immune response by hantavirus. Viral proteins from hantavirus evolving escape strategies involving the inhibition of Interferon (IFN) signaling pathways, the prevent on the host cell apoptosis, the production of microRNA, and the increasing of immune check point molecules.

This review summarizes the molecular and cellular mechanisms on how hantavirus engages several strategies to evade innate immune responses, as illustrated in Figure 1. These mechanisms influence each other. When infection occurs, the cells undergo a series of physiological and pathological responses. Virus infection leads the cells into a stress condition and induces the production of inflammatory cytokines. By regulating target cell death, the virus could also struggle its way to survive. During these processes, the miRNA expression profile is also changed. In turn, the miRNAs regulate the immune response induced by virus infection. The IFN signaling is the end pathway of most processes. The cytokines and surface molecules changes in DCs and monocytes lead to the subsequent cellular mechanisms.

Although the host cells defeat the hantavirus finally, in most cases, the hantaviruses struggled their way to survive. In our perspective, these strategies used to regulate hantavirus immune response negatively could also help maintain a balance between immune activation and inhibition. Further research focusing on various immune responses induced by hantavirus infection will enhance our understanding of hantavirus pathogenesis and develop more effective methods to control infection and fine-tune the activation, strength, and duration of the antiviral immunity.

## Author Contributions

YZ, YW, ZY, WS, and RM wrote the manuscript. MH and TH drew a diagram. RL, YZ, and XW edited, reviewed, and approved the manuscript. All authors contributed to the article and approved the submitted version.

## Funding

This work was supported by the National Natural Science Foundation Grants (Nos. 81772167, 81971563) and Key Research and Development Project of Shaanxi Province (No. 2019ZDLSF02-03).

## Conflict of Interest

The authors declare that the research was conducted in the absence of any commercial or financial relationships that could be construed as a potential conflict of interest.

## Publisher’s Note

All claims expressed in this article are solely those of the authors and do not necessarily represent those of their affiliated organizations, or those of the publisher, the editors and the reviewers. Any product that may be evaluated in this article, or claim that may be made by its manufacturer, is not guaranteed or endorsed by the publisher.
